# Relearning to see: Embodied perception and relational awareness in palliative care

**DOI:** 10.1017/S1478951525101259

**Published:** 2025-12-26

**Authors:** Jose Eric Mella Lacsa

**Affiliations:** Theology and Religious Education, De La Salle University, Malate, Manila, Philippines

Dear Editor,

I read with great interest Henry Bair’s recent article titled *“Color Without Light,”* which argues that true perception extends beyond sight. By listening to patients who have lost their vision, we come to understand that seeing is not confined to the eyes but is instead a deeper, embodied way of experiencing the world, through touch, sound, smell, and attentive awareness (Bair 2025). In his story of a man living with advanced glaucoma, Bair reveals that the experience of color can survive even after light is gone. What the patient describes in temperature, texture, and memory becomes, for the physician, an awakening: the realization that medicine too often forgets the richness of human perception when it limits itself to what can be seen and measured.

This reflection carries a powerful message for those of us in palliative and supportive care. We, too, work daily with people who are gradually losing parts of their world, sight, strength, independence, sometimes even language. One instinct as clinicians is to observe, to measure, to document decline. Yet what Bair’s patient reminds us is that there are other ways of perceiving: ways that depend not on sight but on presence, on listening with the whole body, and on learning to “see” through empathy.

In the Philippines, this way of attending to others finds a natural counterpart in the cultural value of *pakiramdam*, a form of relational sensitivity that means “to feel one’s way into another’s experience” (Guinto and Campoamor-Olegario 2025). It is a kind of knowing that relies less on words and more on shared feeling, the tone of a sigh, the shift of silence, the warmth of a hand. In many Filipino homes where family members serve as caregivers, *pakiramdam* is often what holds care together when medical resources run thin. It turns caregiving into a dialogue of presence, not just a routine of treatment (Tursunova et al. 2022).

Bair’s narrative, when viewed through this lens, becomes more than a story about sight. It is an invitation to relearn how to pay attention. Around the world, medicine tends to privilege the visible, the chart, the scan, the lab result. But the realities of dying and healing often reside in what cannot be easily seen: the quiet trust between patient and caregiver, the rhythm of breath that softens in relief, the texture of calm after pain (Meier and Brawley 2011).

If there is a limitation to Bair’s essay, it lies in its silence about these cultural forms of knowing. Integrating perspectives like *pakiramdam* from the Philippines, *ubuntu* from Africa (Mangena, n.d.), or *ma* from Japan would expand his insight into a truly global conversation about perception and care.

To “see beyond sight” is, ultimately, a moral act. It means recognizing that our capacity to heal is tied to our willingness to be present in all the senses of that word. Light may fade, but attention – humble, embodied, and shared – remains its own form of illumination.
